# Technical variability and safety of sleeve gastrectomy: a nationwide survey of bariatric centers in Poland

**DOI:** 10.20452/wiitm.2025.17954

**Published:** 2025-05-28

**Authors:** Przemysław Sroczyńsk, Piotr Major, Michał R. Janik

**Affiliations:** General Surgery Department, Military Institute of Aviation Medicine, Warszawa, Poland; Second Department of General Surgery, Jagiellonian University Medical College, Kraków, Poland

**Keywords:** complications, operative technique, sleeve gastrectomy, standardization, surgical volume

## Abstract

**INTRODUCTION:**

Sleeve gastrectomy (SG) is the most frequently performed bariatric procedure in Poland. While considered technically straightforward, significant variation exists in the operative technique, which may affect safety outcomes.

**AIM:**

This study aimed to analyze the technical aspects and complication rates of SG across Polish bariatric centers in 2023.

**MATERIALS AND METHODS:**

This is a secondary analysis of a nationwide survey conducted among bariatric centers affiliated with the Metabolic and Bariatric Surgery Chapter of the Association of Polish Surgeons. As many as 54 centers participated, reporting detailed data on SG technique and outcomes. The centers were stratified by annual SG volume using a 50-procedure threshold, corresponding to the suggested learning curve.

**RESULTS:**

A total of 7450 SG procedures were reported. Calibration bougies of 36 French were used by 70% of the centers. Staple line reinforcement was routinely performed in 72.5% of the centers, with oversewing and clipping as the most common methods. Leak tests were performed by 84.3% of the centers, while 66.7% omitted routine drain placement. Postoperative complication rates included bleeding (0.98%), leak (0.33%), reoperation (1.04%), and mortality (0.16%). Although not significant, complication rates were consistently higher in the centers performing fewer than 50 SGs annually.

**CONCLUSIONS:**

SG in Poland is characterized by favorable safety outcomes but notable procedural variability. While most centers follow recommended practices, a lack of standardization persists in key technical areas. Surgical volume may influence complication rates. National guidelines and structured training programs may help harmonize practice and improve outcomes.

## INTRODUCTION

Laparoscopic sleeve gastrectomy (SG) has become the most performed bariatric procedure worldwide and in Poland, owing to its relative technical simplicity, favorable risk profile, and robust outcomes in terms of weight loss and comorbidity resolution.^1^^,^^2^^,^^3^^,^^4^^,^^5^^,^^6^ As reported in our recent national analysis of bariatric surgery in Poland in 2023, SG accounted for 82% of all bariatric procedures performed across 54 centers, underscoring its dominant role in the obesity treatment.^7^

Despite its widespread adoption, significant variation exists in how SG is performed, including differences in calibration bougie size, staple line reinforcement, use of intraoperative leak testing (IOLT), and drain placement. These technical elements, though seemingly minor, may influence perioperative safety and long-term outcomes. In addition, while SG is often perceived as a straightforward procedure, accumulating evidence suggests that its learning curve may impact complication rates, particularly in low-volume centers.^8^

**TABLE 1:** Procedural volume and use of sleeve gastrectomy per center in 2023 in Poland

Parameter	Value
Responding centers, n	54
Bariatric procedures, n	9102
SG procedures, n (%)	7450 (82)
SGs per center, median (range)	97 (15–469)
Centers performing ≥50 SGs per year, n (%)	39 (72.2)
Centers performing ≥200 SGs per year, n (%)	16 (29.6)
Centers performing ≥400 SGs per year, n (%)	4 (5.6)
Centers performing only SGs, n	5
Centers performing only SGs with <50 SGs/year	4

Abbreviations: SG, sleeve gastrectomy

**TABLE 2:** Technical aspects of sleeve gastrectomy

Parameter		Centers, n (%)
Use of 36 Fr bougie		35 (70)
Use of 34 Fr bougie		10 (20)
Use of 32 or 35 Fr bougie		5 (10)
Routine leak test		43 (84.3)
Routine drain placement		17 (33.3)
Staple line reinforcement	None	17 (33.3)
	Oversewing	14 (27.5)
	Clipping	10 (19.6)

Abbreviations: Fr, French (unit of bougie diameter)

**TABLE 3:** Technical practices by center volume (selected examples)

Parameter	≥50< SG/year	<50 SG/year	Total	P value (Fisher test)
Leak test performed, n (%)	29 (80.6)	14 (93.3)	43	0.41
Drain routinely placed, n (%)	12 (33.3)	5 (33.3)	17	>0.99

Abbreviations: see TABLE 1

Understanding the real-world variability in SG practices is essential for identifying areas for standardization, improving training protocols, and ultimately enhancing patient safety. However, few studies have systematically examined these technical aspects and their relationship with postoperative outcomes at the national level.

## AIM

The aim of this study was to identify patterns of practice, assess adherence to international recommendations, and evaluate whether surgical volume correlates with complication rates. This analysis provides valuable insights into the current state of SG implementation and highlights opportunities for optimization of care.

## MATERIALS AND METHODS

This study is a secondary analysis of data collected through a nationwide survey conducted among bariatric surgery centers in Poland. The detailed methodology, including survey design, recruitment strategy, and inclusion criteria, has been described in our earlier publication and remains unchanged in this extended analysis.^7^

In brief, the survey was distributed electronically to all centers affiliated with the Metabolic and Bariatric Surgery Chapter of the Association of Polish Surgeons. Responses were collected via a secure online platform and verified for completeness. Participation in the survey was voluntary but served as a prerequisite for inclusion in the national bariatric directory published by the Association.

Our analysis focused exclusively on SG, the most frequently performed bariatric procedure in Poland. The centers were asked to provide detailed information regarding technical aspects of SG, including the type and size of the calibration bougie, the use of IOLT, staple line reinforcement methods, and routine drain placement. The centers also reported safety outcomes, such as postoperative bleeding, staple line leaks, reoperations, and mortality. Where applicable, the outcomes were stratified by institutional volume, using a threshold of 50 annual SG procedures, based on prior literature identifying this as a learning curve benchmark.^8^

### Statistical analysis

Descriptive statistics were used to summarize the responses. Group comparisons were performed using the χ^2^ test or the Fisher exact test for categorical variables and the Wilcoxon rank-sum test for continuous variables. All analyses were conducted using SAS OnDemand for Academics package (SAS Institute Inc., Cary, North Carolina, United States). A *P* value below 0.05 was considered significant.

### Ethical considerations

In accordance with institutional policies and national regulations, this study did not require ethical review board approval, as no patient-identifying data were collected.

## RESULTS

A total of 54 bariatric centers participated in the nationwide survey, reporting 9102 bariatric procedures performed in 2023. Of these, laparoscopic SG accounted for 7450 operations, representing 82% of all procedures.

**TABLE 4:** Safety outcomes following sleeve gastrectomy

Outcome	Mean, %	SD	Min, %	Max, %	Median, %	Q1, %	Q3, %
Postoperative bleeding	0.98	1.52	0	6.25	0.21	0	1.47
Staple line leak	0.33	0.78	0	4	0	0	0.11
Reoperation	01.04	1.75	0	8.16	0.11	0	1.48
Mortality	0.16	0.88	0	6.25	0	0	0

Abbreviations: Q, quartile

**TABLE 5:** Complication rates by center volume

Complication	≥50 SG/year (n = 39)	<50 SG/year (n = 15)	P value	≥50 SG/year, median (IQR)	<50 SG/year, median (IQR)
Reoperation	0.85 (1)	1.53 (2.87)	0.28	0.41 (0–1.44)	0 (0–2.33)
Bleeding	0.86 (1.05)	1.26 (2.34)	0.26	0.6 (0–1.24)	0 (0–2.33)
Leak	0.3 (0.58)	0.42 (1.16)	0.37	0 (0–0.25)	0
Mortality	0.05 (0.17)	0.45 (1.67)	0.78	0	0

Data are presented as mean (SD) or median (interquartile range).

Abbreviations: see TABLE 1

The median number of SG procedures per center was 97, with a range from 15 to 469. Among the participating centers, 39 (72.2%) performed at least 50 SGs annually, while 15 (27.8%) fell below this threshold. Furthermore, only 16 centers (29.6%) reported performing at least 200 SGs annually, and just 3 centers (5.6%) exceeded 400 SGs per year. Notably, 5 centers reported SG as their only bariatric procedure, and 80% of these performed fewer than 50 SGs annually. Revisional SG was reported by 55.7% of the centers (range, 1–18 procedures), and 13.2% of the centers performed SG combined with fundoplication, with reported volumes ranging from 2 to 8 procedures (TABLE 1).

Out of the 54 centers, 51 provided responses on intraoperative technique. The calibration bougie size varied: 70% of the centers used a 36 French (Fr) bougie, 20% used 34 Fr, and 8% used 32 Fr. A single center (2%) reported using a 35 Fr bougie. A leak test was routinely performed by 84.3% of the centers, while 15.7% reported not using this method. Statistical analysis showed no significant association between the center’s annual SG volume and the use of leak testing (Fisher exact test, *P* = 0.41). Routine drain placement was reported by 33.3% of the centers, while the majority (66.7%) did not leave a drain. No significant differences were observed based on the procedural volume (*P* >0.99). Reinforcement of the staple line was routinely performed in 72.5% of the centers. The most common method was oversewing (27.5%), followed by clipping (19.6%). One-third of the centers (33.3%) reported no reinforcement. The remining centers declared various reinforcement methods (TABLE 2). No significant volume-based differences were identified (TABLE 3).

Postoperative bleeding was reported by 48.1% of the centers, with a mean rate of 0.98% (range, 0%–6.25%). The leak rates were reported by 24.1% of the centers, with a mean of 0.33% (range, 0%–4%). Reoperations were reported in 48.1% of the centers, with a mean reoperation rate of 1.04% (range, 0%–8.16%). Mortality was reported by 9.3% of the centers, with an average postoperative mortality rate of 0.16% (range, 0%–6.25%; TABLE 4).

Safety outcomes did not differ significantly based on the center volume (high vs low) with reoperation rate of 0.85% vs 1.53% (*P* = 0.28), bleeding rate of 0.86% vs 1.26% (*P* = 0.26), leak rate of 0.3% vs 0.42% (*P* = 0.37), and mortality of 0.05% vs 0.45% (*P* = 0.78; TABLE 5).

### DISCUSSION

This nationwide analysis provides a comprehensive overview of current practices related to laparoscopic SG in Poland, with a specific focus on procedural variability and safety outcomes. With data collected from 54 bariatric centers reporting 7450 SG procedures in 2023, this study offers one of the most detailed insights into technical standards and complication profiles of this operation in a European national context.

Stratification of the centers, based on the annual volume threshold of 50 SG procedures, proposed as a proxy for overcoming the learning curve, showed that nearly three-quarters of the Polish bariatric units meet this benchmark. Nevertheless, approximately 28% of the centers performed fewer SGs, and interestingly, the majority of the centers that reported SG as their sole bariatric procedure belonged to this lower-volume group. These findings may raise concerns regarding the comprehensiveness of training, procedural selection, and outcomes in such institutions, particularly as low procedural volume has been associated with higher complication rates in previous studies.^9^**^,^**^10^

Despite SG being a standardized procedure in theory, the present study highlights significant heterogeneity in key intraoperative practices. Most centers used a 36 Fr calibration bougie, in line with general recommendations, yet a subset of units adopted narrower bougies.^11^ This observation aligns with previous findings suggesting that variations in bougie size may influence outcomes, such as weight regain or insufficient weight loss, potentially impacting the need for revisional surgery.^12^ This variation reflects an ongoing debate in the literature regarding the optimal bougie size. Some studies have suggested that narrower bougies (30–32 Fr) may be associated with greater weight loss without increasing the risk of early or late complications, as demonstrated in the SOS (Swedish Obese Subjects) study.^13^ In contrast, a recent systematic review analyzing over 4900 patients reported that bougie sizes of at least 40 Fr were associated with significantly lower leak rates than smaller bougies, without compromising weight loss outcomes.^14^ These conflicting findings underscore the complexity of establishing a universal standard. It is also worth noting that in Poland calibration bougies larger than 36 Fr are rarely available.

Reinforcement of the staple line remains debatable, and practices among Polish centers vary widely. While oversewing and clipping were the most commonly reported reinforcement strategies, one-third of the centers reported not reinforcing the staple line at all. This heterogeneity corresponds to continued debate in the literature concerning the clinical benefits of staple line reinforcement. A recent network meta-analysis of 17 randomized controlled trials involving nearly 4000 patients found that suture reinforcement was associated with significantly lower risks of bleeding, staple line leak, and overall complications as compared with no reinforcement.^15^

Supporting these findings, a recent retrospective analysis from our center demonstrated that continuous suturing of the staple line was associated with a complete absence of bleeding complications, whereas 7.1% of patients in the clipping group experienced postoperative hemorrhage. While suturing increased operative time, its potential benefit in terms of hemostasis may be clinically relevant, especially in high-risk patients.^16^ Taken together, these findings highlight both the heterogeneity of current practices and the need for further high-quality studies to determine the most effective and efficient reinforcement technique in SG.

Leak testing was performed routinely by over 84% of the centers, which aligns with best-practice guidelines^10^ aimed at intraoperative complication prevention. However, the clinical value of IOLT remains controversial. A large analysis of the MBSAQIP database involving over 265 000 patients found no significant differences in postoperative leak rates, mortality, or reoperation between those who underwent IOLT and those who did not, suggesting limited benefit in routine use.^17^ A systematic review and meta-analysis including nearly 470 000 SG cases even reported a slightly higher rate of staple line leaks in the IOLT group (0.38%) than the non-IOLT group (0.31%), although IOLT was associated with a significantly lower rate of postoperative bleeding. These findings suggest that IOLT may be more useful in detecting or preventing hemorrhagic complications rather than reducing the risk of a leak.^18^ In contrast, another study evaluating the methylene blue test found high specificity and a negative predictive value of 99%, indicating it may help reliably rule out leaks when positive findings are absent.^19^ Taken together, these conflicting results highlight that while IOLT is widely practiced, there is no clear consensus on its role in preventing postoperative leaks, and its use should likely be tailored to intraoperative findings and patient-specific risks.

Similarly, routine drainage was omitted by most centers, reflecting a global trend toward a more selective approach based on intraoperative risk assessment. Data from the MBSAQIP registry show a marked decline in drain use during SG—from over 20% in 2015 to under 14% in 2017.^20^ Studies have shown that routine drain placement is associated with higher rates of anastomotic leaks, reoperations, and overall morbidity, without improving early detection or outcomes.^20^**^,^**^21^ The current practice in Polish centers aligns with this evidence-based approach.

Importantly, no significant associations were found between procedural volume and the use of reinforcement, drainage, or leak testing.

The reported safety outcomes were generally favorable and consistent with international benchmarks. In high-volume centers, the mean postoperative bleeding rate was below 1%, while leak and reoperation rates remained under 0.5% and 1%, respectively. The results are consistent with large registry data. An MBSAQIP analysis of over 175 000 SG cases reported a 0.6% bleeding rate, with hemorrhage significantly increasing reoperation, readmission, and 30-day mortality.^22^ In contrast, a single-center study focusing specifically on hemorrhagic complications reported a higher rate of 4%, highlighting how reported incidence may vary depending on data source and study design.^23^

Mortality was extremely rare, with an average rate of 0.16% across all institutions. This is slightly higher than the 0.05% mortality rate for SG reported in a recent meta-analysis of over 3.6 million patients, but remains within the range considered safe for bariatric surgery.^24^ The small discrepancy may reflect differences in volume, patient selection, or reporting. Overall, these findings confirm that SG is a low-risk procedure with favorable safety outcomes. However, it is concerning that the maximum mortality rates reported by individual centers deviated markedly from the overall average. This underscores the need to foster a culture of systematic outcome monitoring, particularly in the context of patient safety. Identifying the centers with outlier complication or mortality rates is essential to ensuring targeted support, continuous quality improvement, and ultimately, better outcomes for patients undergoing bariatric procedures.

Although the differences in complication rates between high- and low-volume centers did not reach significance, trends observed in the data—particularly higher reoperation, bleeding, leak, and mortality rates in lower-volume institutions—suggest potential clinical relevance. These findings emphasize the importance of surgical experience and procedural volume in optimizing patient safety, even if definitive conclusions require studies with larger sample sizes.

The observed procedural variability underscores the need for greater standardization of surgical technique in SG. While individualization of practice is expected to some extent, the implementation of national or society-endorsed guidelines could help harmonize care delivery.

The limited use of complex or hybrid techniques, such as SG with fundoplication, reported by a minority of centers, likely reflects the investigational nature of this procedure.^25^ Although of growing interest, it is not yet widely endorsed by major surgical societies as a standard treatment option. Similarly, the low number of revisional SGs may be attributed to the narrow and specific indications for performing SG as a revisional procedure, which significantly limit its routine use in this context.^26^**^,^**^27^

### Limitations

This study is limited by its survey-based methodology, which is susceptible to reporting bias and incomplete data. Not all centers provided full responses to all technical questions, and outcome measures, such as bleeding, leak, and mortality, were self-reported without independent validation. Future studies should aim to integrate registry-based data and clinical audits to enhance reliability and granularity.

## CONCLUSIONS

This nationwide study highlights considerable variability in the technical execution of laparoscopic SG across bariatric centers in Poland. While most institutions follow generally accepted principles—such as the use of a 36 Fr calibration bougie and routine leak testing—significant heterogeneity persists in areas such as staple line reinforcement and postoperative drain use. Notably, one-third of the centers do not reinforce the staple line, and practices vary widely even among high-volume centers.

Safety outcomes for SG in Poland are favorable and align with international benchmarks. The average rates of postoperative bleeding, leak, reoperation, and mortality remained low, particularly in high-volume centers. Although significance was not reached, clinically relevant trends suggest that lower-volume centers may be associated with higher complication rates.

These findings underscore the need for greater procedural standardization, particularly in technical aspects that may influence outcomes. The results also reinforce the importance of maintaining adequate surgical volume to ensure optimal patient safety. Future initiatives should focus on developing national guidelines, promoting structured training pathways, and implementing a centralized registry to monitor outcomes and guide continuous quality improvement in bariatric surgery.

## JOURNAL INFORMATION

Videosurgery and Other Miniinvasive Techniques is an official journal of the Videosurgery Foundation.
